# Sinapic Acid Ameliorates Cadmium-Induced Hepatotoxicity: Modulation of Oxidative Stress, Inflammation, and Apoptosis

**DOI:** 10.3390/biomedicines13051065

**Published:** 2025-04-28

**Authors:** Yomna A. Farahat, Norhan M. El-Sayed, Reem M. Hazem, Eman T. Mehanna, Asmaa Radwan

**Affiliations:** 1 Department of Pharmacology & Toxicology, Faculty of Pharmacy, Suez Canal University, Ismailia 41522, Egypt; pgs.14210162@pharm.suez.edu.eg (Y.A.F.); reem_ahmed@pharm.suez.edu.eg (R.M.H.); asmaa_elsayed@pharm.suez.edu.eg (A.R.); 2 Department of Biochemistry, Faculty of Pharmacy, Suez Canal University, Ismailia 41522, Egypt

**Keywords:** cadmium, hepatotoxicity, sinapic acid, oxidative stress, inflammation, apoptosis

## Abstract

**Background/Objectives**: Cadmium (Cd) is a harmful metal commonly used in industry. Numerous clinical diseases, including osteomalacia, testicular damage, renal and hepatic failure, and pulmonary edema, are associated with Cd exposure. The current study evaluated the protective effect of Sinapic acid (SA) against Cd-induced hepatotoxicity by investigating different mechanistic pathways interfering with Cd-related liver injury. **Methods**: Forty rats were randomly assigned to four groups as follows; group 1 served as negative control and received saline, group 2 received saline for 14 days and CdCl_2_ (3.5 mg/kg IP) as a single dose on day 14, groups 3 and 4 were treated with SA (20, 40 mg/kg PO), respectively, for 14 days and injected with CdCl_2_ (3.5 mg/kg IP) on day 14. Serum was collected to evaluate liver function. Liver samples were collected for histopathological examination and the assessment of markers related to oxidative stress, inflammation, and apoptosis. **Results**: Acute Cd administration elevated liver enzymes and induced pathological changes in liver specimens, with the concurrent release of inflammatory markers and reduced antioxidant capabilities. Pretreatment with SA improved liver function and Cd-induced histopathological changes and elevated the activities of antioxidant enzymes. SA ameliorated inflammation, as evidenced by decreased expression of NF-κB, TNF-α, TLR-4, and COX-2, iNOS, and IL-1β levels along with suppression of mTOR, JNK, ERK, BAX, and Bcl-2. **Conclusions**: The present data suggest that SA represents a promising protective agent against Cd-induced hepatic injury by attenuating oxidative stress, inflammation, and apoptosis.

## 1. Introduction

Cadmium (Cd) is a hazardous heavy metal often found in low environmental concentrations. In recent years, Cd has become increasingly prevalent due to its industrial and agricultural use [1]. The risk of human exposure is rising as Cd penetrates the food chain and persists in the environment, with a long half-life (25–30 years). It primarily enters the human body through inhalation and ingestion [2]. Cd poisoning affects several biological systems, including the neurological, cardiovascular, gastrointestinal, immunological, and reproductive systems [3]. It primarily accumulates in the liver and kidneys. Acute and chronic Cd exposure contributes to liver diseases such as hepatitis, fatty liver disease, cirrhosis, and hepatocellular carcinoma [4].

Intoxication by Cd disrupts normal cellular function by displacing essential metals and generating reactive oxygen species (ROS). This oxidative stress overwhelms the body’s natural antioxidant defenses, depleting crucial enzymes like catalase (CAT), superoxide dismutase (SOD), and glutathione (GSH), resulting in widespread cellular damage [5].

Exposure to Cd triggers an inflammatory response that attracts white blood cells and adhesion molecules. The recruited cells generate cytokines such as IL-1β, TNF-α, IL-6, and IL-8, which enhance immune cell activation and migration. Inflammatory cells produce various reactive species, exacerbating oxidative stress. Furthermore, ROS produced indirectly by inflammatory cells, in addition to direct oxidative stress, amplify signaling pathways that upregulate proinflammatory gene expression [3].

Additionally, Cd induces apoptosis through both intrinsic and extrinsic pathways. It disrupts mitochondrial membranes, leading to the release of cytochrome C and activation of caspases, or it binds to death receptors in the Fas/FasL pathway. Both pathways ultimately result in hepatic cell apoptosis and elevated liver enzyme levels [6].

Sinapic acid (SA) is a hydroxycinnamic acid that belongs to the phenolic acids family and contains bioactive carboxylic acids. SA is an orally bioavailable phytochemical found in various plants, including spices, citrus and berry fruits, vegetables, cereals, and oilseed crops [7]. Due to its neuroprotective [8], antihyperglycemic [9], antibacterial, and anticancer [10] properties, SA has demonstrated a wide range of potential therapeutic benefits.

The potent antioxidant properties of SA and its metabolites have gained increasing attention. SA scavenges harmful ROS such as DPPH^∙^, O^∙−^, and ^∙^OH, reduces lipid peroxidation, and minimizes oxidative damage [11]. Additionally, SA exhibits anti-inflammatory activity by suppressing several inflammatory mediators [12,13,14]. Furthermore, SA has demonstrated the ability to inhibit caspase-3 [13], suggesting a potential anti-apoptotic effect. The antioxidant, anti-inflammatory, and anti-apoptotic properties of SA enable it to serve as a protective agent against environmental hepatotoxins such as arsenic [15] and other hepatic insults such as methotrexate [16].

Given that oxidative stress is a central mechanism in Cd-induced liver damage, the antioxidant capability of SA is hypothesized to mitigate hepatotoxicity by neutralizing ROS and stabilizing antioxidant defenses. Therefore, the current study aimed to explore the possible protective role of SA against various mechanisms of Cd-induced hepatotoxicity including inflammation and apoptosis.

## 2. Materials and Methods

### 2.1. Drugs and Chemicals

Sinapic acid was obtained from Sigma-Aldrich (D7927-5G, Sigma-Aldrich, St. Louis, MO, USA), and cadmium chloride (CdCl_2_) with a purity of 96% was provided by the Analytical Department, Faculty of Pharmacy, Mansoura University (Mansoura, Egypt). Both CdCl_2_ and SA were dissolved in isotonic saline.

### 2.2. Animals

Forty mature male Wistar rats weighing between 150 and 200 g were obtained from the Egyptian Organization for Biological Products and Vaccines (Vacsera, Giza, Egypt). The rats took one week to acclimate before the study began. Water and food were provided ad libitum. Rats were housed in cages with 12 h of light and 12 h of darkness, with an interior temperature of 23 ± 2 °C and a relative humidity of 30–35%. The ARRIVE guidelines were followed in the current study and the research protocol was approved by the research ethics committee of the Faculty of Pharmacy, Suez Canal University, Ismailia, Egypt (license number 202207MA1).

### 2.3. Experimental Design

Four groups of ten animals each [17,18,19] were randomly allocated as follows: group 1 served as the negative control and received 0.5 mL isotonic saline per oral (PO) for 14 days and intraperitoneal (IP) saline on day 14; group 2 served as the Cd-intoxicated group, which received isotonic saline PO for 14 days and a single IP dose of CdCl_2_ (3.5 mg/kg) [20] on day 14; groups 3 and 4 represented the SA treated groups, which received SA at doses of 20 and 40 mg/kg/day PO, respectively [15,16], for 14 days and CdCl_2_ (3.5 mg/kg) IP on day 14. The choice of sample size and SA doses was based on the previous literature.

Blood samples were withdrawn within 24 h after CdCl_2_ administration, and serum was separated by centrifuging for 15 min at 5000 rpm. The serum was then refrigerated at −20 °C for additional examination. Rats were euthanized under thiopental sodium (30 mg/kg) anesthesia. The livers were dissected and cut into two halves. The first half was preserved in 10% formaldehyde for histological and immunohistochemical investigations, while the other half was preserved at −80 °C for biochemical analysis. To reduce bias, the study was performed using a blind approach, ensuring that researchers involved in data collection and outcome evaluation were unaware of the animal group assignments.

### 2.4. Assessment of Liver Weight Index and Liver Function

Both animal weight and liver weight were determined. The liver weight index was calculated using the following equation: liver weight index = (liver weight)/(body weight) × 100.

Alanine aminotransferase (ALT) (Cat. No: AL 1031, Biodiagnostic-Egypt, Mansoura, Egypt) and aspartate aminotransferase (AST) (Cat. No: AS 1061, Biodiagnostic-Egypt, Mansoura, Egypt) were assessed colorimetrically.

### 2.5. Evaluation of Inflammation and Necrosis in Histopathology

Histopathological scoring was conducted based on the presence and extent of inflammatory infiltration and necrosis. Inflammatory cell infiltration was graded as follows: scattered inflammatory cells (score 1), focal aggregations (score 2), and diffuse infiltration (score 3). Necrosis was scored as: absent (score 0), mild (score 1), marked (score 2), and severe (score 3) [21].

### 2.6. Assessment of Cadmium and Calcium in the Liver

ELISA kits were used to measure the liver levels of Cd (Cat. No: MBS3809624, MyBioSource, San Diego, CA, USA) and Calcium (Ca) (Cat. No: DICA-500, Bioassay systems, Hayward, CA, USA) using the sandwich ELISA technique according to the manufacturer’s instructions.

### 2.7. Assessment of Oxidative Stress Biomarkers Levels

As instructed by the manufacturer, the levels of malondialdehyde (MDA) (Cat. No. MBS268427, MyBioSource, San Diego, CA, USA), GSH (Cat. No. E02G0367 BlueGene Biotech, Shanghai, China), SOD (Cat. No: MBS036924, MyBioSource, San Diego, CA, USA), CAT (Cat. No: MBS006963, MyBioSource, San Diego, CA, USA), and heme oxygenase-1 (HO-1) (Cat. No. ADI-EKS-810A, Enzo, Zandhoven, Belgium) were measured using ELISA kits.

### 2.8. Assessment of Gene Expression by Quantitative Real Time PCR

The expression of the genes coding for nuclear factor kappa-light chain-enhancer of activated B cells (NF-κB), tumor necrosis factor-alpha (TNF-α), nuclear factor erythroid 2-related factor 2 (Nrf2), toll like receptor-4 (TLR-4), and p53 were examined using the quantitative real-time PCR method. Following the manufacturer’s instructions, total RNA was isolated from four distinct frozen liver tissue samples per group using the ABT Total and Micro RNA mini extraction kit (Applied Biotechnology, Ismailia, Egypt). A NanoDrop spectrophotometer (Thermo Fisher Scientific, Waltham, MA, USA) was used to measure the extracted RNA’s concentration and purity. ABT 2X one step qRT-PCR Mix (SYBR) high ROX (Applied Biotechnology, Ismailia, Egypt) was used to assess the expression of the target genes in liver tissue. Glyceraldehyde 3-phosphate dehydrogenase gene (*GAPDH*) was used as the endogenous control. Table 1 shows the primers and annealing temperatures. The PCR reaction involved the following components: 1 µL from each of the forward and reverse primers (10–15 picomoles), 10 µL 2X one step qRT-PCR Mix, 100 to 1000 ng RNA (≈4 µL) extract, and up to 20 µL nuclease-free water. The cycle included reverse transcription for 20 min at 42 °C, before three minutes of heating to 95 °C, followed by 45 cycles of 15 s of denaturation at 95 °C, 30 s of annealing, and 30 s of extension at 72 °C. The StepOnePlusTM Real-Time PCR thermal cycling apparatus (Applied Biosystems, Carlsbad, CA, USA) was used. To guarantee the validity of the data, each sample was performed in duplicate. Additionally, a negative control for PCR with no template was used to prevent DNA contamination. ∆∆Ct and fold change values were computed, where the fold change = 2^−∆∆Ct^ (Table 1).

### 2.9. Assessment of Inflammatory Markers Levels

Using sandwich ELISA kits, the levels of cyclooxygenase-2 (COX-2) (Cat. No.: MBS020734, MyBioSource, San Diego, CA, USA), inducible nitric oxide synthase (iNOS) (Cat. No. MBS723326, MyBioSource, San Diego, CA, USA), and interleukin-1β (IL-1β) (Cat. No. MBS825017, MyBioSource, San Diego, CA, USA) were measured.

The levels of c-Jun N-terminal kinase (JNK) (Cat. No. RTFI00924, AssayGenie, Dublin, Ireland), extracellular signal- regulated kinase (ERK) (Cat. No. LS-F27419, LSBio, Newark, CA, USA), 5′ adenosine monophosphate-activated protein kinase (AMPK) (Cat No. MBS765897, MyBioSource, San Diego, CA, USA), and mammalian target of rapamycin (mTOR) (Cat No:LS-F17553, LSBio, Newark, CA, USA) were assessed using the sandwich ELISA kit following the manufacturer’s instructions.

### 2.10. Western Blot Analysis of LC3

Liver was homogenized with 1 ml of Trifast reagent (Peqlab, VWR International, Radnor, PA, USA) using a glass-Teflon homogenizer. The protein pellet was vacuum-dried for 5–10 min, then dissolved in 1% SDS at 50–100 °C to enhance solubilization. The solution was subsequently centrifuged at 10,000× *g* for 10 min at 4 °C. After protein quantification in the supernatant using the Bradford assay, samples were loaded onto 10% SDS-PAGE, and electrophoresis was performed at 75 volts through the stacking gel followed by 125 volts for approximately 2 h. Separated proteins were transferred to a Hybond™ nylon membrane (GE Healthcare, Chicago, IL, USA) via a TE62 Standard Transfer Tank (Hoefer Inc., Bridgewater, MA, USA). Membrane protein blocking was performed by incubating the samples in 2–5% nonfat dry milk and Tris-buffered saline with Tween 20 (TBST) for 1 h at room temperature to prevent nonspecific binding. The membranes were then incubated overnight at 4 °C with primary antibodies for LC3 I/II (Cat No. BS67340, bioworlde, Nanjing, China) and β-actin (Cat No. ab8227, Abcam, Waltham, MA, USA). The blots were washed with at least five changes of TBST for 30–60 min at room temperature, then incubated with an HRP-conjugated polyclonal secondary antibody (Cat No. HAF007, R&D Systems, Minneapolis, MN, USA) for 1 h at room temperature. Afterward, the membranes were rinsed again with TBST. Protein band intensity was analyzed using TotalLab Analysis Software Ver. 1.0.1 (GelDoc-It, UVP, Upland, CA, USA) and normalized to β-actin as the housekeeping gene.

### 2.11. Immunohistochemical Assessment of Apoptotic Markers

The protein expression of B-cell lymphoma-2 (Bcl-2) and Bcl-2-associated X protein (BAX) were determined using immunohistochemistry. On adhesive slides, five µm tissue slices were cut, deparaffinized, and then rehydrated in distilled water. Heat-induced epitope retrieval was applied to tissue sections and the primary anti-BAX antibody, mouse monoclonal IgG2b κ BAX antibody, (sc-7480, Santa Cruz Biotechnology, Inc., Dallas, TX, USA) was then incubated at a dilution of 1:200, and primary anti-Bcl-2 antibody, rabbit polyclonal IgG antibody, (26593-1-AP, Proteintech, Planegg-Martinsried, Germany) at a dilution of 1:200 for one hour at room temperature. After washing, peroxidase blocking was performed, followed by detection using an HRP-labelled secondary detection kit (BioSB, Santa Barbara, CA, USA), as the manufacturer instructed. Negative control slides were made by avoiding primary antibody incubation. Positive expression was measured as the mean area percentage of positive staining in each group. The positively stained area was brown in color and was observed against the negatively stained region. Finally, color intensity was estimated using an image analyzer (Image J program Version 1.51).

### 2.12. Statistical Analysis

The data were analyzed using SPSS (version 26) and images were generated by Graphpad Prism 10. Data normality was assessed by the Shapiro–Wilk test, and multi-group analysis was performed by the one-way analysis of variance (ANOVA) test, followed by the Bonferroni post hoc multiple comparison test. The results were expressed as mean ± standard deviation (SD). Statistical significance was considered for *p* < 0.05 in all comparisons between study groups. Histopathological hepatic injury scores were presented as median and quartiles and statistically assessed using the Kruskal–Wallis test, followed by Dunn’s multiple comparisons test.

## 3. Results

### 3.1. Effect of SA on Cd Accumulation in Liver Tissues

**Figure 1:**
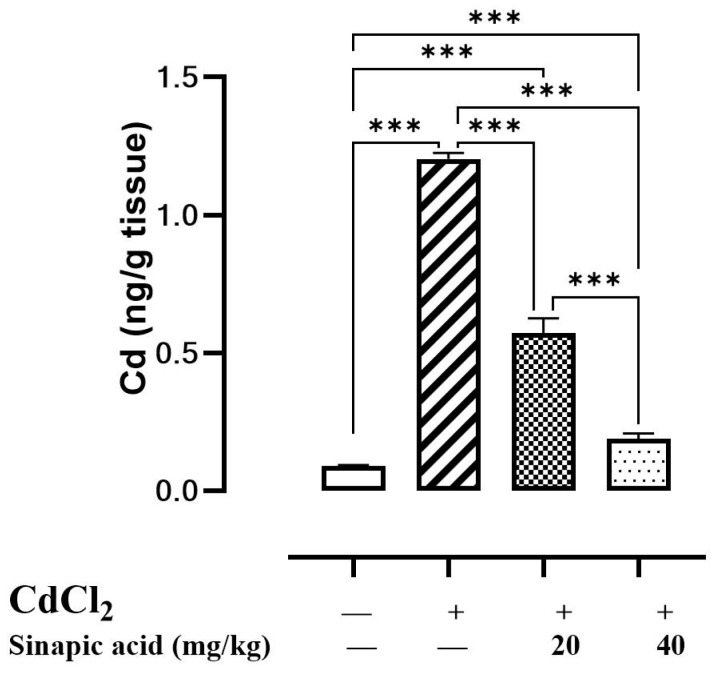
Effect of SA on CdCl_2_ concentration in liver. Data are presented as mean ± SD. *** represents significant difference at *p* ˂ 0.001 using one-way ANOVA test followed by Bonferroni test for multiple comparisons.

Upon the evaluation of hepatic Cd levels, it was found to be over ten times greater in Cd-intoxicated animals than in control animals, suggesting hepatic accumulation. Interestingly, pretreatment with different doses of SA significantly (*p* < 0.05) reduced hepatic Cd levels in a dose-dependent manner (Figure 1).

### 3.2. Effect of SA on Liver Function

The Cd-intoxicated group showed a significant increase in ALT and AST levels compared to the control group, while the SA pretreated groups showed a significant (*p* < 0.05) decrease in both enzymes in a dose-dependent manner. The liver weight index showed an insignificant difference among groups (Table 2).

### 3.3. Effect of SA on Histopathological Examination

**Figure 2:**
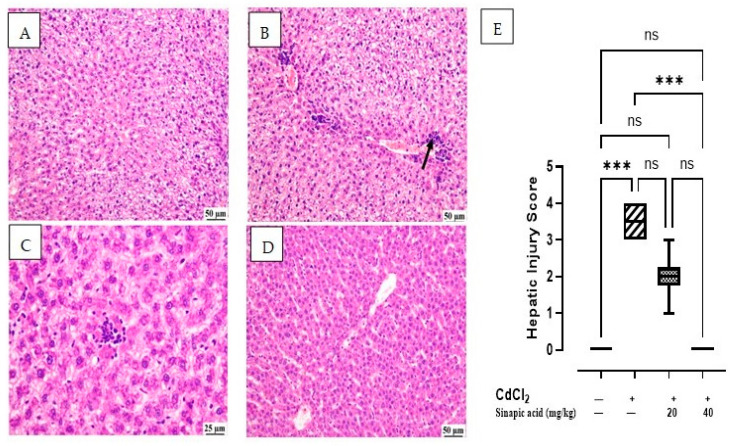
Histopathological examination of liver: (**A**) control group showing normal hepatic parenchyma, (**B**) CdCl_2_-intoxicated group showing multifocal inflammatory cells infiltration (arrow) with vacuolation of hepatic parenchyma, (**C**) SA 20 mg/kg group showing random fewer inflammatory cells infiltration, (**D**) SA 40 mg/kg group showing apparently normal hepatic parenchyma, and (**E**) hepatic injury score in different groups. Data are expressed as median and quartiles, *** indicates a significant difference between group pairs at *p* < 0.001 and ns indicates no significant difference at (*p* < 0.05) using the Kruskal–Wallis test followed by post hoc Dunn test.

The microscopic examination of the control group showed normal hepatic parenchyma; hepatocytes of both centrilobular and portal areas were normal. On the contrary, the CdCl_2_-intoxicated group exhibited multifocal areas of hepatocellular necrosis and marked vacuolation in hepatocytes with mononuclear inflammatory cell infiltration. This acute inflammation was accompanied by polymorphonuclear neutrophil infiltration. Some of the examined sections showed mild portal fibroplasia. Moderate vacuolation was noticed in the SA low-dose group in some examined sections with limited portal fibroplasia; several sections revealed normal hepatic parenchyma. Normal hepatic parenchyma was observed in the examined liver sections from the SA high-dose group. The hepatic injury score in Figure 2E showed significant (*p* < 0.05) improvement in inflammation and necrosis in the histological picture of livers treated with a high dose of SA compared to the CdCl_2_-intoxicated group (Figure 2).

### 3.4. Effect of SA on Lipid Peroxidation and Antioxidant Enzymes

A state of oxidative stress was observed in the CdCl_2_-intoxicated group, as evidenced by a remarkable rise in MDA levels and a reduction in levels of antioxidant markers (GSH, SOD, and CAT) compared to control group. Treatment with SA significantly (*p* < 0.05) decreased the MDA level and restored the GSH, SOD, and CAT pool.

Both Nrf2 gene expression and HO-1 levels were significantly (*p* < 0.05) diminished by the Cd insult. Different doses of SA attained a higher gene expression of Nrf2 and HO-1 level in a dose-dependent manner (Table 3).

### 3.5. Effect of SA on Ca Levels in Liver Tissue

**Figure 3:**
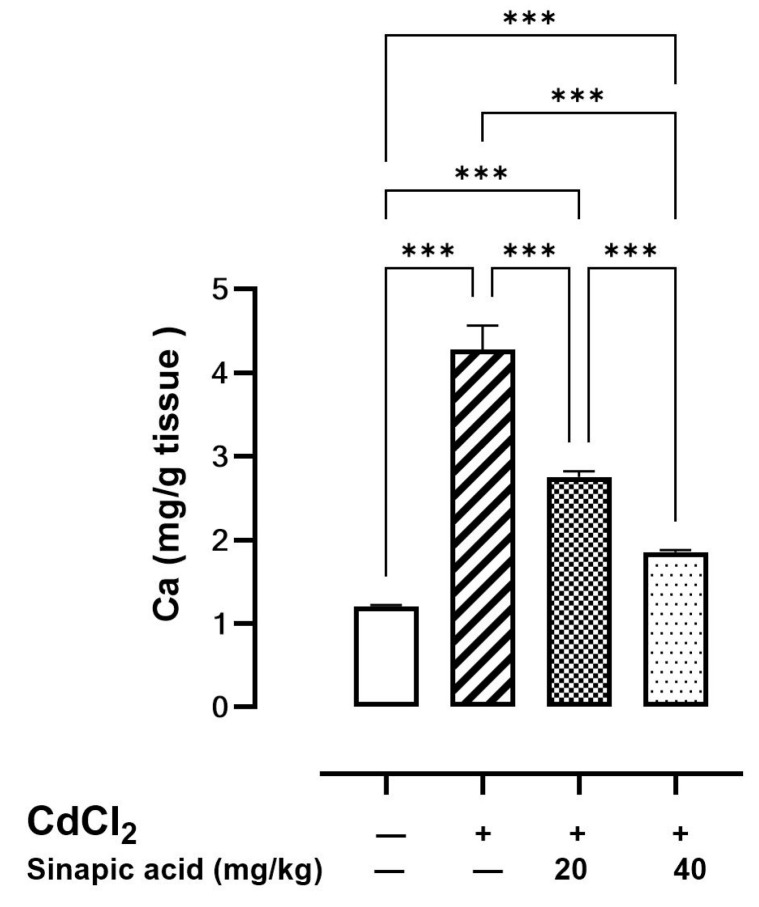
Effect of SA on Ca levels in liver. Values are expressed as mean ± SD. Significant difference is considered at *p* < 0.05. *** indicates significant difference at *p* < 0.001 using one-way ANOVA test followed by Bonferroni test for multiple comparisons.

The cellular Ca level was elevated in the Cd-intoxicated group compared to the control group. Interestingly, SA treatment with different doses caused a significant reduction in the Ca level in a dose-dependent manner (Figure 3).

### 3.6. Effect of SA on Inflammatory Markers

**Figure 4:**
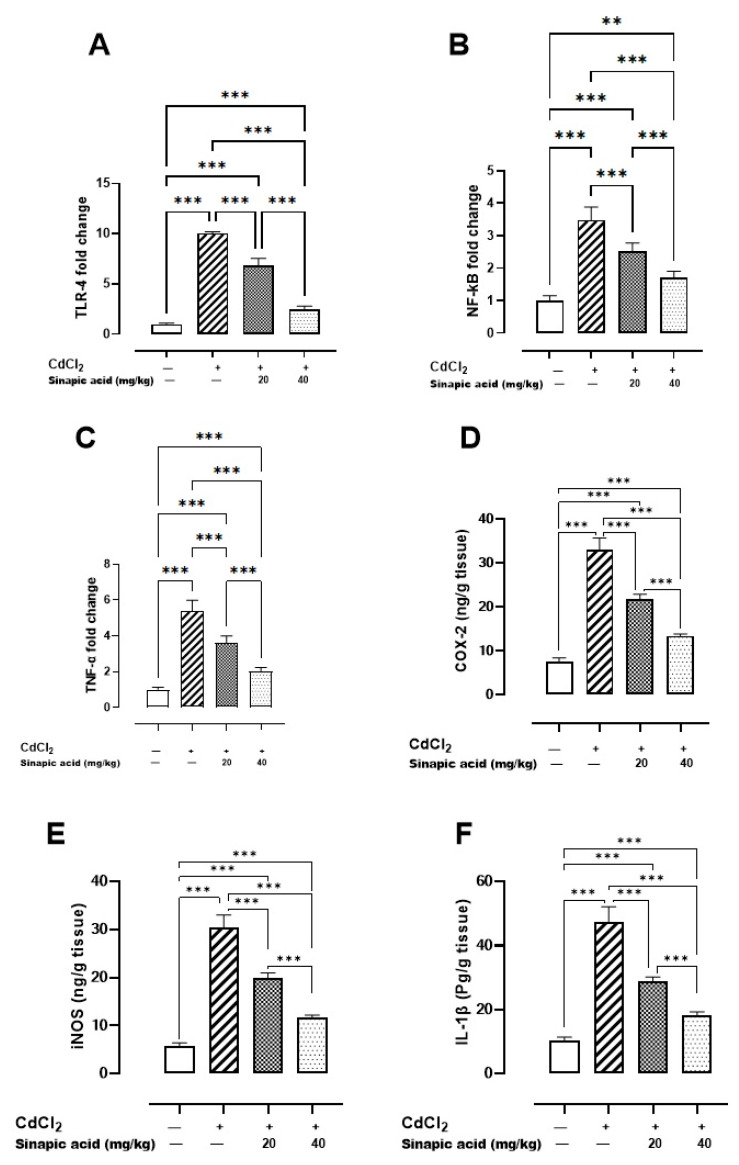
Effect of SA on inflammatory markers: (**A**) TLR-4 gene expression, (**B**) NF-κB gene expression, (**C**) TNF-α gene expression, (**D**) COX-2 level, (**E**) iNOS level, and (**F**) IL-1β level. Values are expressed as mean ± SD. Significant difference is considered at *p* < 0.05. ** indicates significant difference at *p* < 0.01, *** indicates significant difference at *p* < 0.001 using one-way ANOVA test followed by Bonferroni test for multiple comparisons.

The Cd-intoxicated group displayed a notable increase in all inflammatory markers compared to the control group. SA treatment decreased TLR-4, NF-κB, and TNF-α gene expression and attenuated IL-1β, COX-2, and iNOS levels in a dose-dependent manner (Figure 4).

### 3.7. Effect of SA on Autophagy Markers

**Figure 5:**
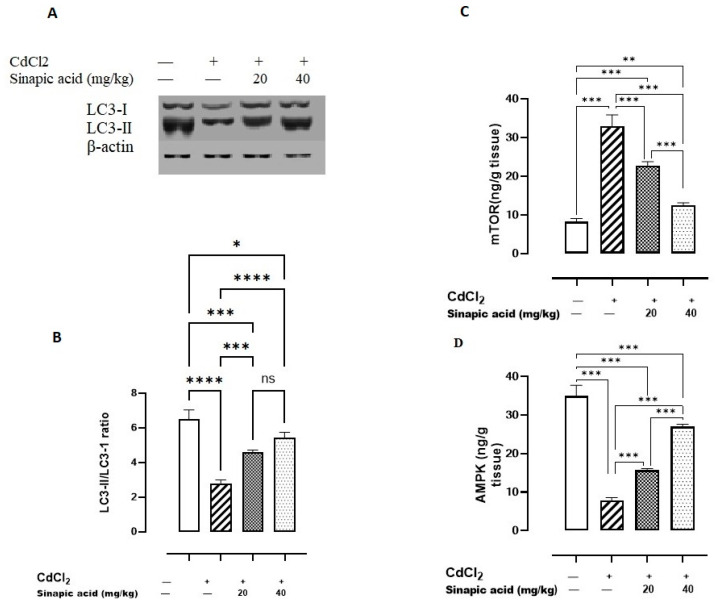
Effect of SA on autophagy markers. (**A**) LC3-I/II expression, (**B**) LC3-II/LC3-I ratio, (**C**) mTOR, and (**D**) AMPK. Data are presented as mean ± SD. A significant difference is considered at *p* < 0.05. * indicates a significant difference at *p* < 0.05. ** indicates a significant difference at *p* < 0.01, *** indicates a significant difference at *p* < 0.001, **** indicates a significant difference at *p* < 0.0001, ns indicates no significant difference at *p* < 0.05 using one-way ANOVA test followed by Bonferroni test for multiple comparisons.

Exposure to Cd significantly (*p* < 0.05) lowered AMPK levels and the LC3-II/LC3-I ratio and amplified mTOR levels compared to the control group. Variable doses of SA showed a significant (*p* < 0.05) rise in AMPK levels and LC3-II/LC3-I ratio and a reduction in mTOR levels in a dose-dependent manner (Figure 5).

### 3.8. Effect of SA on MAPK Pathways

**Figure 6:**
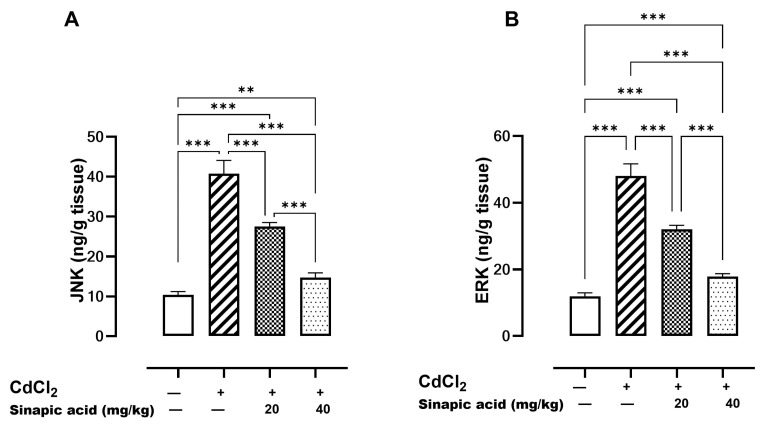
Effect of SA on MAPKs. (**A**) JNK and (**B**) ERK. Data are presented as mean ± SD. Significant difference is considered at *p* < 0.05. Significant difference is considered at *p* < 0.05. ** indicates significant difference at *p* < 0.01, *** indicates significant difference at *p* < 0.001 using one-way ANOVA test followed by Bonferroni test for multiple comparisons.

Mitogen-activated protein kinases (MAPKs) such as JNK and ERK are essential modulators for apoptosis. A significant (*p* < 0.05) elevation in both JNK and ERK levels was observed in the Cd-insulted group, which gradually resolved with SA administration (Figure 6).

### 3.9. Effect of SA on Markers of Apoptosis

**Figure 7:**
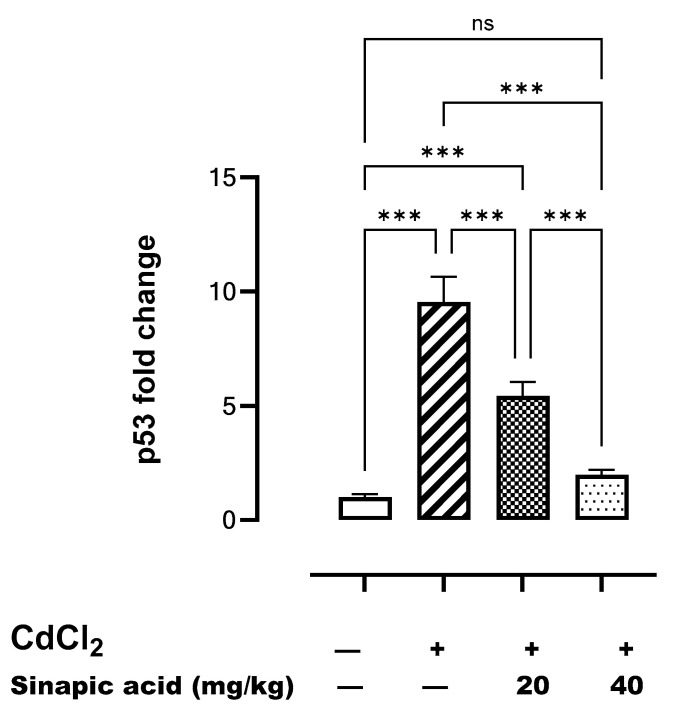
Effect of SA on *p53* gene expression in liver. Data are presented as mean ± SD. *** indicates significant difference at *p* < 0.001, ns indicates no significant difference at *p* < 0.05 using one-way ANOVA test followed by Bonferroni test for multiple comparisons.

The gene expression of *p53* was significantly (*p* < 0.05) higher following Cd insult compared to the control. However, *p53* gene expression gradually decreased with SA administration in a dose-dependent manner (Figure 7).

**Figure 8:**
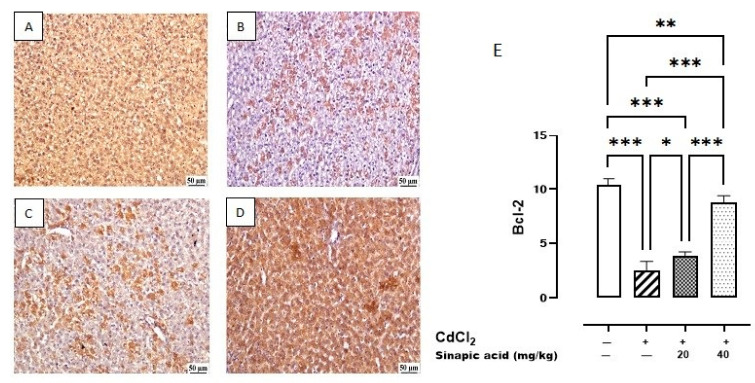
Photomicrograph of liver Bcl-2 immune staining. (**A**) Control group showing higher Bcl-2 expression, (**B**) Cd-intoxicated group showing weak Bcl-2 expression, (**C**) SA 20 mg/kg group showing moderate Bcl-2 expression, (**D**) SA 40 mg/kg group showing higher expression, and (**E**) percentage of expression of Bcl-2 in different groups. Data are presented as mean ± SD. * indicates significant difference at *p* < 0.05. ** indicates significant difference at *p* < 0.01, *** indicates significant difference at *p* < 0.001 using one-way ANOVA test followed by Bonferroni test for multiple comparisons.

The control group showed the highest expression of Bcl-2 in the hepatic parenchyma. A lower expression was observed in Cd-intoxicated livers and livers treated with a low dose (20 mg/kg) of SA. Meanwhile, a higher expression was determined in the hepatic tissue-treated group with a high dose (40 mg/kg) of SA (Figure 8).

**Figure 9:**
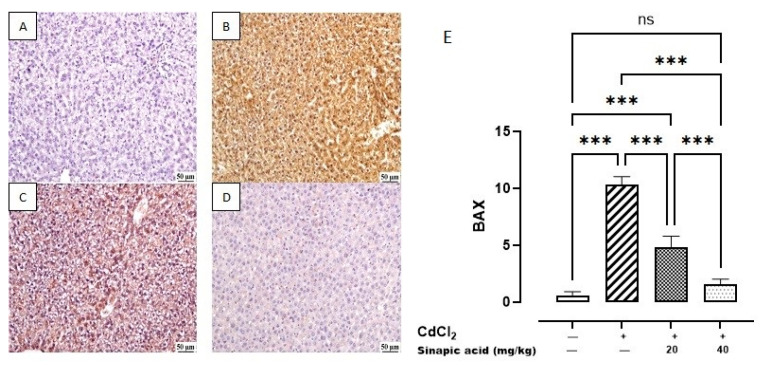
Photomicrograph of liver BAX immune staining: (**A**) control group showing limited BAX expression, (**B**) Cd-intoxicated group showing intense BAX expression, (**C**) SA 20 mg/kg group showing moderate BAX expression, (**D**) SA 40 mg/kg group showing limited expression, and (**E**) BAX expression percentage of different groups. Data are presented as mean ± SD. *** indicates significant difference at *p* < 0.001, ns indicates insignificant difference at *p* < 0.05 using one-way ANOVA test followed by Bonferroni test for multiple comparisons.

The control group revealed a minimal expression of BAX in the hepatic parenchyma. Meanwhile, a higher expression was noticed in the Cd group. A significant decrease was observed in SA-treated livers with the high dose (40 mg/kg) compared to SA-treated livers with the low dose (20 mg/kg) (Figure 9).

## 4. Discussion

Although Cd is used in a wide variety of sectors, it is a hazardous heavy metal. It is considered a hepatotoxin because of its capacity to alter the histology of the liver and impair liver function [4]. The objective of this study was to assess the efficacy of SA in mitigating the hepatotoxicity induced by Cd.

In the present investigation, there was no significant difference in the liver weight index among the groups. This can be explained by the fact that liver collection occurred a day after Cd administration, meaning there was insufficient time for organ remodeling. This is in accordance with prior research that showed no noticeable difference in the liver weight index among animals poisoned with Cd [22].

Regarding Cd distribution, the current study revealed that Cd toxicity resulted in a substantial increase in hepatic Cd levels, suggesting hepatic accumulation. It was reported that the ionic form of Cd tends to accumulate in the liver. This is due to the fact that it can cross the hepatic cell membrane by binding with the Fe and Zn transporter and binding to voltage-gated Ca channels. The non-ionic form of Cd can bind to metallothionein produced in the liver to form the Cd-Metallothionein (Cd-MT) complex, which facilitates hepatocellular entrance through receptor-mediated endocytosis [4,23].

Upon entering hepatic cells, Cd binds to intracellular metallothionein, forming a Cd-MT complex that maintains a low concentration of free Cd ions. However, in instances of elevated Cd exposure, the binding capacity of metallothionein is surpassed, resulting in an excess of free Cd ions that intoxicates cells, ultimately leading to cell death [24]. It was also demonstrated that ROS and reactive nitrogen species (RNS) generated by Cd can destabilize the (Cd-MT) complex, resulting in elevated concentrations of Cd within cells [25].

Treatment with SA, a natural product with antioxidant properties, in the current study showed a suppression of Cd hepatic accumulation. This can be attributed to its ability to reduce ROS and RNS production, resulting in less dissociation of the Cd-MT complex [25].

In the present study, it was observed that liver enzymes increased in association with Cd insult, indicating liver injury and cell death. Cd toxicity results in liver injury through both necrosis and programmed cell death, which liberates the cellular content and increases serum levels of liver enzymes ALT and AST [22,26]. Furthermore, liver injury was confirmed through histopathological examination, which demonstrated hepatocellular necrosis, multifocal inflammatory cell infiltration, and portal congestion. This aligns with a previous study showing tissue necrosis, apoptosis, hyperplasia, and the enlargement of the hepatic sinus and hilum in livers damaged by Cd [27].

Treatment with SA in the current study showed a protective effect, against Cd-induced hepatotoxicity, evidenced by decreased hepatic Cd accumulation, enhanced liver function, decreased serum liver enzymes, and improved liver histopathology, which appeared to be normal in the higher dose. These results are consistent with previous studies, which reported that SA has a hepatoprotective effect against hepatic toxicants such as paracetamol [28].

In the current study, Cd insult led to high MDA levels, demonstrating lipid peroxidation along with an extreme reduction in antioxidant GSH, SOD, and CAT, reflecting the loss of a defense mechanism against oxidation. These data collectively represent a state of oxidative stress. Oxidative stress was proven to be the main player contributing to Cd toxicity as ROS accumulation disrupts cellular equilibrium, resulting in damage to numerous cell components [3]. Moreover, Cd possesses an oxidative capacity by participating in the Fenton reaction [29] or by binding to sulfhydryl groups in certain proteins, such as GSH [25].

The current study revealed that the Nrf2/HO-1 pathway was inhibited in Cd-intoxicated rats. This can be explained by the ability of both Nrf2 and HO-1 to serve as additional protective mechanisms against oxidative stress, and they were inhibited in a dominant state of oxidation. The Nrf2/HO-1 pathway acts as a defense mechanism against oxidative stress, inflammation, and apoptosis, and it is crucial for the prevention of liver injury [30]. Following these findings, the relationship between Cd toxicity and Nrf2 inhibition was established, as the inhibition of Nrf2 leads to an increase in ROS generation and a worsening of oxidative stress [26].

Treatment with SA mitigated oxidative stress by decreasing lipid peroxidation and MDA levels, while simultaneously replenishing the antioxidant enzyme pool. It was reported that SA has effective antioxidant properties, as it scavenges free radicals such as O_2_∙− and •OH and decreases lipid peroxidation [10]. Similarly, SA attenuated Cd-induced oxidative stress in kidneys by inhibiting MDA levels and improving catalase activity [25]. Moreover, SA improved the defensive Nrf2/HO-1 pathway. This improvement in oxidative stress and restoring balance between ROS and antioxidant enzymes positively impacted all other pathways of Cd toxicity. SA-associated Nrf2 enhancement was previously established against cisplatin-induced oxidative stress in nephrotoxicity [25].

The current data indicated a substantial increase in hepatic Ca levels in rats intoxicated with Cd compared to normal rats. Both ROS and Ca are regarded as significant weapons that Cd employs to manipulate the survival mechanism of cells and induce apoptosis and necrosis. The interplay between ROS and Ca signaling is considered bidirectional, since ROS can regulate cellular Ca signaling, while Ca signaling is essential for ROS production. Consequently, elevated Ca levels trigger ROS-producing enzymes and the synthesis of free radicals [31]. SA pretreatment corrected the disrupted Ca level, which had an impact on ROS production and apoptosis. The reduction in both ROS and Ca is the main key of SA prophylactic therapy against Cd toxicity.

The present findings also revealed that Cd toxicity induced inflammation through stimulating TLR-4, NF-κB, and TNF-α expression in addition to increasing levels of IL-1β, COX-2, and iNOS. TLR-4 is an essential regulator for cytokine production and immune responses [32]. It was established that TLR-4 activates NF-κB through the MYD88 pathway, which increases NF-κB expression. This stimulation of NF-κB expression triggers an inflammatory cascade, as it increases TNF-α production and upregulates iNOS and COX-2, which are responsible for production of IL-1β [33].

In addition, Nrf2 inhibition and ROS accumulation are potent NF-κB activators, whereas elevated NF-κB is recognized as a Nrf2 suppressor [4]. Moreover, high levels of TLR-4 can potentiate ROS production as TLR-4 activates NADPH oxidase, which in turn facilitates the transmembrane transport of electrons and production of ROS [34]. TLR-4 induces high levels of iNOS and subsequently produces NO. The generation of NO worsens oxidative stress by suppressing SOD activity and promoting MDA formation. SA attenuated Cd-induced inflammation as it suppressed TLR-4, NF-κB, and TNF-α expression and consequently reduced the levels of inflammatory cytokines COX-2, iNOS, and IL-1β. The anti-inflammatory effect of SA was reported in inflammatory bowel disease, as it decreased inflammatory markers such as NF-κB, TNF-α, and IL-1β [11]. Likewise, the effect of SA on NF-κB and Nrf2 was observed in cardiomyopathy in diabetic rats [35].

The present data revealed that Cd insult was associated with a reduction in the LC3-II/I ratio, which suggests the inhibition of autophagy. This finding aligns with a previous study on Cd-induced hepatic toxicity, which linked Cd exposure to dysregulated autophagy. The presence of elevated ROS and Ca levels due to Cd exposure disrupts normal autophagy by inhibiting autophagosome production, directing cells to apoptosis [36].

The current study demonstrated that Cd toxicity is linked to the suppression of AMPK, a positive regulator of autophagy, and the elevation of mTOR levels, which additionally indicates autophagy inhibition. This can be attributed to TLR-4 activation, which phosphorylates mTOR, thereby reducing the production of unc-51-like autophagy activating kinase 1 (ULK-1), a critical component of autophagic vesicles, and suppressing autophagy [34]. Moreover, mTOR activation can inhibit Bcl-2 and elevate caspase-3/7 levels through the JNK pathway to accelerate apoptosis [37]. AMPK is also a key cellular energy regulator, which preserves mitochondrial function and counteracts oxidative stress [38] and inflammation [39] under metabolic stress.

Treatment with SA improved autophagy markers by upregulating the AMPK level and LC3 expression while suppressing mTOR levels, thereby promoting autophagy as a survival mechanism. Autophagy induction by SA was previously reported against hepatoma as SA increases LC3 and Beclin-1 levels [40]. Additionally, the SA upregulation of AMPK enhanced cellular homeostasis, replenished antioxidant enzymes, and suppressed oxidative stress and inflammation. Furthermore, SA pretreatment downregulated mTOR, which subsequently inhibited apoptosis and promoted cell survival. This agrees with the previous literature, as SA has been shown to elevate AMPK levels and downregulate mTOR levels, thereby reducing inflammation and inhibiting apoptosis in intestinal fibrosis [41].

In the current study, apoptosis was induced in hepatic cells exposed to Cd, as evidenced by increased *p53* gene expression, decreased anti-apoptotic Bcl-2 expression, and increased proapoptotic BAX expression. The activation of *p53* triggers the transcription of several genes that cause cell cycle arrest and induce apoptosis by upregulating proapoptotic BH3-only proteins such as *p53* upregulated modulator of apoptosis (*PUMA*) and phorbol-12-myristate-13-acetate-induced protein 1 (*Noxa*), which in turn activate BAX and counteract the anti-apoptotic effects of Bcl-2 [42].

The overload of Ca is also another trigger for apoptosis, as it increases mitochondrial membrane permeability. The resulting irreversible increase in the permeability of the inner mitochondrial membrane to small molecules, along with mitochondrial depolarization, amplified ROS production, and outer mitochondrial membrane rupture, lead to the release of pro-apoptotic factors into the cytosol, thereby activating the intrinsic apoptotic signaling pathway [43]. Furthermore, Ca accumulation can help cytochrome C exit the mitochondria to the cytoplasm, initiating apoptosis [44].

Apoptotic cell death was repressed by SA pretreatment, as it inhibited *p53* gene expression, decreased BAX expression, and enhanced anti-apoptotic Bcl-2 expression. Matching with this, the anti-apoptotic effect of SA was assessed against ulcerative colitis, as it inhibited BAX and stimulated anti-apoptotic Bcl-2 expression [45].

Regarding the effect of Cd toxicity on the MAPK pathway, an elevation of both ERK and JNK were observed. Sustained ERK activation was reported to be a consequence of the high ROS level in Cd toxicity and was linked to cell cycle arrest and Ca-induced apoptosis [46]. The elevated JNK level was linked to apoptosis induction, as activated JNK can translocate to nucleus, activate c-Jun, and increase the expression of pro-apoptotic genes. Furthermore, activated JNK translocates to the mitochondria and phosphorylate BH3-only family of Bcl-2 proteins to antagonize the anti-apoptotic activity of Bcl-2. In addition, JNK can stimulate the release of cytochrome C from the mitochondrial inner membrane, promoting the formation of apoptosomes consisting of cytochrome C, caspase-9, and apoptotic protease activating factor-1 (Apaf-1). This complex initiates the activation of a caspase-9-dependent caspase cascade [47]. JNK also has been previously documented to have a role in the development of inflammation in interstitial cystitis, since its activation enhances the release of IL-1β and TNF-α [40].

Recalling the above-mentioned data, it is supposed that Cd toxicity induces the apoptosis of hepatic cells through a complex interaction between many pathways, including excessive Ca and ROS production, the inhibition of the AMPK/mTOR pathway, activation of the MAPK pathway, and direct simulation of apoptotic factors.

In the present study, a high level of MAPKs, JNK, and ERK, was modulated by SA pretreatment and in turn inhibited BAX expression and enhanced Bcl-2 to suppress apoptosis. This agrees with a previous study that proved SA enhanced inflammation in rat chondrocytes through the downregulation of p38 MAPK, JNK, and ERK [14].

Comparative studies between SA and the conventional hepatoprotective drug Silymarin indicated that both Silymarin and SA restored antioxidant enzymes such as GSH, SOD, and CAT [48]. Additionally, both drugs elevated Nrf2 and HO-1 activity [49] to alleviate oxidative stress. Regarding inflammation, Silymarin, similarly to SA, showed a significant reduction in inflammatory mediators such as TLR-4 [50], NF-κB [51], TNF-α [52], COX-2 [51], and IL-1β [50]. Furthermore, Silymarin was able to suppress IL-6 and transforming growth factor beta (TGF-β) [51]. Both Silymarin and SA affected the AMPK/mTOR pathway by enhancing AMPK and reducing mTOR levels [53], triggering hepatocellular survival. Considering apoptosis, Silymarin was found to suppress pro-apoptotic JNK, and subsequently BAX, while replenishing anti-apoptotic Bcl-2 [48], which was also proven with SA treatment.

## 5. Conclusions

This study proved that SA ameliorated Cd-induced liver toxicity by restoring cell survival mechanisms such as antioxidants and the AMPK/mTOR pathway. Furthermore, SA treatment inhibited ROS, inflammation, and cellular death by apoptosis via multiple mechanisms: regulating the NF-κB pathway and the subsequent inflammatory mediators, modulating MAPK/ JNK/ ERK signaling, and modifying the BAX /Bcl-2 ratio. However, the limitations of this study include the lack of SA toxicity assessment. Overall, SA demonstrates potential hepatoprotective effects but requires further validation.

## Figures and Tables

**Table 1:** Primers and annealing temperatures used in real-time PCR.

GenBank Accession No.	Gene	Primers	Annealing Temperature
NM_199267.2	*RELA* (coding for NF-κB subunit)	Forward: 5′-CAATGGCTACACAGGACCA-3′ Reverse: 5′-CACTGTCACCTGGAACCAGA-3′	51 °C
NM_012675.3	*TNF* (coding for TNF-α)	Forward: 5′-TCTACTGAACTTCGGGGTGATCG-3′ Reverse: 5′-TGATCTGAGTGTGAGGGTCTGGG-3′	55 °C
NM_031789.3	*NFE2L2* (coding for Nrf2)	Forward: 5′-CTCTCTGGAGACGGCCATGACT-3′ Reverse: 5′-CTGGGCTGGGGACAGTGGTAGT-3′	58 °C
NM_019178.2	*TLR4*	Forward: 5′-TTTATTCAGAGCCGTTGGTG-3′ Reverse: 5′-CAGAGGATTGTCCTCCCATT-3′	50 °C
NM_001429995.1	*p53*	Forward: 5′-ACCGCCGACCTATCCTTACC-3′	55 °C
		Reverse: 5′-TCTTCTGTACGGCGGTCTCTC-3′	
NM_017008.4	*GAPDH*	Forward: 5′-ATGACTCTACCCACGGCAAG-3′ Reverse: 5′-GATCTCGCTCCTGGAAGATG-3′	52 °C

**Table 2:** Effect of SA on liver function.

	Control	CdCl_2_ + Saline	SA (20 mg/kg) + CdCl_2_	SA (40 mg/kg) + CdCl_2_
Liver weight index	3.3 ± 0.0036	3.5 ± 0.0053	3.7 ± 0.0053	3 ± 0.0044
AST	59.15 ± 5.61	141.22 ± 19.55 ^a^	98.92 ± 9.97 ^ab^	69.12 ± 5.9 ^bc^
ALT	40.11 ± 5.56	112.48 ± 15.34 ^a^	83.28 ± 9.28 ^ab^	56.29 ± 6.02 ^bc^

Effect of SA on liver weight, ALT and AST. Values are expressed as mean ± SD. Superscript letters indicate significant difference at *p* < 0.05 using one-way ANOVA test followed by Bonferroni test for multiple comparisons, ^a^ represents significant difference vs. control group, ^b^ represents significant difference vs. CdCl_2_-intoxicated group, ^c^ represents significant difference vs. SA (20 mg/kg) group.

**Table 3:** Effect of SA on oxidative stress markers.

	Control	CdCl_2_ + Saline	SA (20 mg/kg) + CdCl_2_	SA (40 mg/kg) + CdCl_2_
MDA	6.9 ± 0.75	30.1 ± 2.1 ^a^	19.9 ± 0.9 ^ab^	12.7 ± 0.75 ^abc^
GSH	66.1 ± 4.6	15.1 ± 1.7 ^a^	26.9 ± 1.2 ^ab^	39.7 ± 1.9 ^abc^
SOD	56.9 ±3.3	14.7 ± 1.5 ^a^	26 ± 1.1 ^ab^	38.6 ± 1.9 ^abc^
CAT	51.3 ± 2.9	12.6 ± 1.3 ^a^	22.1 ± 1.1 ^ab^	31.8 ± 1.6 ^abc^
Nrf2	1 ± 0.1	0.39 ± 0.05 ^a^	0.62 ± 0.07 ^ab^	0.85 ± 0.1 ^abc^
HO-1	29.55 ± 1.26	6.55 ± 0.63 ^a^	12.62 ± 0.46 ^ab^	22.13 ± 0.88 ^abc^

Effect of SA on oxidative stress markers MDA, GSH, SOD, CAT, and Nrf2/HO-1 pathway. Values are expressed as mean ± SD. Superscript letters indicate significant difference at *p* < 0.05 using one-way ANOVA test followed by Bonferroni test for multiple comparisons, ^a^ represents significant difference vs. control group, ^b^ represents significant difference vs. Cd-intoxicated group, ^c^ represents significant difference vs. SA (20 mg/kg) group.

## Data Availability

Data are available within the article.

## Abbreviations

The following abbreviations are used in this manuscript:

Cd	Cadmium
ROS	Reactive oxygen species
CAT	Catalase
SOD	Superoxide dismutase
GSH	Glutathione
IL-1β	Interleukin 1 beta
TNF-α	Tumor necrosis factor alpha
SA	Sinapic acid
FasL	Fas ligand
DPPH	2,2-diphenyl-1-picrylhydrazyl
PO	Peroral
IP	Intraperitoneal injection
SD	Standard deviation
ANOVA	Analysis of variance
ALT	Alanine aminotransferase
AST	Aspartate aminotransferase
MDA	Malondialdehyde
Nrf2	nuclear factor erythroid 2–related factor 2
HO-1	Heme oxygenase 1
Ca	Calcium
TLR-4	Toll-like receptor 4
NF-κB	nuclear factor kappa-light chain-enhancer of activated B cells
iNOS	Inducible nitric oxide synthase
COX-2	Cyclooxygenase-2
AMPK	Adenosine monophosphate-activated protein kinase
mTOR	Mammalian target of rapamycin
LC3	Microtubule-associated protein 1A/1B-light chain 3
MAPK	Mitogen-activated protein kinases
JNK	Jun N-terminal kinase
ERK	Extracellular signal-regulated kinase
Bcl-2	B cell lymphoma-2
BAX	Bcl-2–associated X protein
Cd-MT	Cadmium-Metallothionein
RNS	Reactive nitrogen species
NO	Nitric oxide
ULK-1	Unc-51-like kinase 1
Apaf-1	Apoptotic protease activating factor 1
ELISA	Enzyme-linked immunosorbent assay
RT PCR	Real-Time polymerase chain reaction
TGF-β	Transforming growth factor beta
